# Extricating Sex and Gender in Air Pollution Research: A Community-Based Study on Cardinal Symptoms of Exposure

**DOI:** 10.3390/ijerph10093801

**Published:** 2013-08-22

**Authors:** Tor H. Oiamo, Isaac N. Luginaah

**Affiliations:** 1 Social Science Centre, Department of Geography, Western University, 1151 Richmond Street, London, ON N6A 5C2, Canada; 2 Department of Geography, Western University, London, ON N6A 5C2, Canada; E-Mail: iluginaa@uwo.ca

**Keywords:** gender, sex, air pollution, environmental health, occupational exposure, allergic disease, LUR, CART

## Abstract

This study investigated sex and gender differences in cardinal symptoms of exposure to a mixture of ambient pollutants. A cross sectional population-based study design was utilized in Sarnia, ON, Canada. Stratified random sampling in census tracts of residents aged 18 and over recruited 804 respondents. Respondents completed a community health survey of chronic disease, general health, and socioeconomic indicators. Residential concentrations of NO_2_, SO_2_, benzene, toluene, ethylbenzene and *o/m/p*-xylene were estimated by land use regression on data collected through environmental monitoring. Classification and Regression Tree (CART) analysis was used to identify variables that interacted with sex and cardinal symptoms of exposure, and a series of logistic regression models were built to predict the reporting of five or more cardinal symptoms (5+ CS). Without controlling for confounders, higher pollution ranks increased the odds ratio (OR) of reporting 5+ CS by 28% (*p* < 0.01; Confidence Interval (CI): 1.07–1.54). Females were 1.52 (*p* < 0.05; CI: 1.03–2.26) times more likely more likely to report 5+ CS after controlling for income, age and chronic diseases. The CART analysis showed that allergies and occupational exposure classified the sample into the most homogenous groups of males and females. The likelihood of reporting 5+ CS among females was higher after stratifying the sample based on occupational exposure. However, stratifying by allergic disease resulted in no significant sex difference in symptom reporting. The results confirmed previous research that found pre-existing health conditions to increase susceptibility to ambient air pollution, but additionally indicated that stronger effects on females is partly due to autoimmune disorders. Furthermore, gender differences in occupational exposure confound the effect size of exposure in studies based on residential levels of air pollution.

## 1. Introduction

Sex and gender, which can be operationalized as biology and culture, have the potential to confound results in environmental health studies of air pollution. The terms “sex” and “gender” have often been used interchangeably to simply differentiate men and women, but furthermore Clougherty [1] suggests that study design or analytical approaches limit the extrication of sex and gender effects in studies where different meanings are assigned. Regarding studies on health effects of air pollution general, residence-based pollutant exposure models are limited by lack of temporal variability associated with daily activities, while longitudinal studies are often limited by spatially aggregated measures of exposure. Even less is understood about how gender as a cultural construct interacts with these limitations, therefore physiological systems have hitherto provided the most likely sources of observed differences in effects of pollution on males and females.

Effects of criteria air contaminants on the respiratory system are well documented, and several studies have reported differing outcomes for males and females [2,3]. Meta-analyses of respiratory effect modification of sex and gender are difficult to complete due to varying exposure mixes, outcomes and analytic techniques, however, more studies report stronger effects among women [1]. Causal mechanisms for this remain unclear though experimental and clinical studies have corroborated this assertion and offer at least a partial explanation. For example, lung particulate matter deposition characteristics differ among men and women and the relative amount of deposition is greater in women due to higher flow rates [4]. As measured by fatigue and pulmonary function, women are slightly more sensitive than men to effects of 2-propanol and *m*-xylene vapours, which are among volatile organic compounds (VOCs) released by petrochemical processing and combustion engine exhaust [5].

Cyclical fluctuations in sex hormones during the reproductive period of life increases the prevalence rates of atopic disease in women, though rates are lower compared to males during childhood and after menopause [6]. Females have a lower risk of developing asthma in their childhood, equal risk during adolescence, and higher risk during early adulthood, which is attributed to smaller airway caliber and hormonal factors [7], while air pollution is known to exacerbate asthma [8]. Interestingly, immunoglobulin E (IgE) serum levels are higher in males throughout life, but sex specific differences in allergen sensitivity are inconsistent [6]. Air pollutants can trigger IgE responses [9], and a population-based study in London, England observed increased primary care consultations for allergic rhinitis in association with outdoor air pollution [10].

However, sex and gender differences in susceptibility to air pollution are expressed beyond the respiratory system. Transdermal exposure pathways along with the irritant properties of pollution upon the skin itself are important as women exhibit higher rates of skin disease [11]. There is also evidence in support of hormonal status attenuating the effect of particulate matter on heart disease in women less than 60 years of age [12]. Complementing our understanding of biologically plausible mechanisms are environmental health studies that demonstrate significant associations between air pollution exposure profiles and sociocultural, socioeconomic and demographic factors [13,14], all of which can be influenced by gender. Furthermore, stress can potentiate or attenuate impacts of air pollution, and both hormonal and sociocultural characteristics of sex and gender, respectively, can moderate stress levels [15].

Taken together, the aforementioned and broad range of outcomes and moderating factors associated with air pollution effects on health produce a complex challenge to public health policy makers, practitioners and administrators. As previously mentioned, both study design and analytical approaches are important considerations when attempting to disentangle the closely related, but distinct constructs of sex and gender. Therefore, this study used a novel analytical approach to determine the influence of sex and gender on susceptibility to short-term effects of air pollution. Recursive partitioning and regression modeling were utilized to examine the characteristics of sex and gender that contribute to the commonly observed stronger effect of air pollution on women.

## 2. Methods

### 2.1. Sampling and Exposure Assessment

This study uses a combination of spatial, environmental and survey data collected in and around Sarnia’s “Chemical Valley” in Ontario. The study population is exposed to air pollution from a combination of industrial and mobile sources. Forty per cent of the chemical processing facilities in Canada are located in Chemical Valley, and high volumes of traffic are associated with a busy border crossing at the Blue Water Bridge connecting Sarnia with Port Huron. MI, USA. Studies of hospital admissions standardized by age show significantly higher admission rates in Sarnia than both London and Windsor, which are also located in Southern Ontario [16], and residential levels of air pollution are associated with primary health care utilization [14]. The area has garnered widespread attention because the proportion of male births in the Aamjiwnaang First Nation community near Sarnia declined significantly between 1984 and 2003 [17]. The close proximity of this community to Chemical Valley and consequent exposure to compounds that can cause endocrine disruption and compromise reproductive health have been put forth as potential causes [17,18]. Furthermore, a high incidence of occupational diseases of the respiratory and neurological systems has also been identified in the area, along with the highest rates of mesothelioma in Canada [17,18,19].

A stratified sampling procedure was used to recruit participants from each census tract in Sarnia. Canadian Viewpoint Ltd., a survey company in Toronto, ON, conducted the survey in September and October of 2005 using a computer assisted telephone interview system. The sample represented approximately 1% of the Sarnia population, yielding a total of 804 respondents with a response rate of 62%. The Non-Medical Research Ethics Board at Western University approved the study.

During administration of the survey over a two-week period in October, several ambient air pollutants including nitrogen dioxide (NO_2_), SO_2_ and the VOCs benzene, toluene, ethylbenzene, *m/p*- and *o*-xylene (BTEX) were monitored at 39 locations throughout the city of Sarnia [20,21]. Land use regression (LUR) was utilized to model the ambient NO_2_, SO_2_ and total BTEX concentrations. For a comprehensive review of LUR models for exposure assessment in epidemiologic studies see Hoek *et al*. [22]. To avoid multicollinearity as a result of high correlation between the different pollutants, a ranking method was used to create a single pollution factor. Each case (n = 804) was assigned 3 ranks based on their estimated levels of residential ambient exposure to SO_2_, NO_2_, and BTEX [23]. The 3 ranks were summed to give each case a single measure of exposure relative to all other cases, and these sums were in turn ranked from high to low. This produced a uniform distribution that was normalized using the inverse cumulative distribution function. Table 1 describes the correlation between SO_2_, NO_2_, BTEX and the pollution factor.

**Table 1:** Descriptive sample characteristics ^+^ and bivariate statistics for categorical and continuous variables in relation to sex/gender and pollution factor.

	Sample (n = 804)	Females (n = 440)	Males (n = 364)	Sex Difference	Pollution Factor
Categorical Variables	%	%	%	*Pearson* χ^2^	*Mann-Whitney Test* (*2-tailed. z*)
5+ Cardinal Symptoms	19.3	21.9	16.2	4.44 *	−2.77 **
Occupational Exposure	46.3	27.4	68.8	137.22 ***	−1.44
Hay Fever or Allergies	29.0	35.1	21.7	17.09 ***	−0.52
Asthma	9.5	10.5	8.4	1.02	−2.29 *
Cancer	4.6	3.5	5.8	2.45	−1.54
Kidney Disease	4.8	4.0	5.7	1.45	−0.08
Skin Condition	13.9	16.9	10.4	7.27 **	−1.17
Hypertension	22.8	22.5	23.1	0.05	−0.73
Heart Disease	7.9	5.9	10.3	5.24 *	−1.44
Low Income	10.5	10.6	10.3	0.01	−5.39 ***
**Continuous Variables**	μ_X_	μ_X_	μ_X_	*Independent t-Test*	*Pearson r (2-tailed*)
NO_2_ (ppb)	13.85	13.75	13.96	1.86	0.84 ***
SO_2_ (ppb)	3.18	3.17	3.19	0.14	0.76 ***
BTEX (mg/m^3^)	3.76	3.73	3.81	0.77	0.77 ***

^+^ Standardized to Sarnia by age; * p < 0.05; ** p < 0.01; *** p < 0.001.

### 2.2. Outcome Measurement

Health outcomes of air pollution exposure with demonstrated sex and/or gender differences include daily mortality [24], respiratory hospitalization [3], peak respiratory flow [25], and odour annoyance [26]. We chose Cardinal Symptom (CS) reporting as our outcome because it provides a broad measure of potential short-term and irritant properties of air pollution [27]. The variable used in the analysis is a composite representation of respondents’ experience of the following symptoms at least once a month during the summer preceding exposure assessment: Burning or discomfort urinating; coughing not related to a cold; earaches; hives or skin rashes; irritated, sore or red eyes; nausea; nosebleeds; runny or stuffy nose not related to a cold; sinus congestion not related to a cold; sore throat not related to a cold, and; wheezing or other trouble breathing. Cardinal Symptoms were analyzed as a binary (0–4 *vs*. 5+) variable because the number of symptoms and their distribution were inappropriate as an ordinal measure or a linearizing transformation. The cut-off was determined by maximizing the area under the Receiver Operator Characteristics (ROC) Curve for NO_2_, SO_2_ and BTEX and the pollution factor. This identified the binary classification with highest sensitivity to different concentrations and a mixture of the pollutants. The areas under the ROC curve were all significantly more than 0.5 when comparing 0–4 to 5+ symptoms, which indicated that the association between this number of cardinal symptoms and NO_2_, SO_2_, BTEX and the pollution factor was not random. This relationship is possibly owed to the unlikely case of 5 or more cardinal symptoms being due solely to seasonal allergies or comorbidities.

### 2.3. Gendered Stratification Variables

Previous studies have identified stronger effects of air pollution on females by analyzing samples stratified by sex [2]. This method is more informative than adjustment with sex as a covariate and can identify broad differences in associations between the health outcome and the sexes, but it can also obscure sources of variability relating to exposure and susceptibility [1]. Therefore, this study follows an approach suggested by Clougherty [1] and stratifies the sample according to gendered variables in the context of exposure. Classification & Regression Tree (CART) analysis is a recursive partitioning method and was utilized to detect variables that interacted with reporting of 5+ CS. The CART algorithm uses the Gini improvement measure, which in this study was maximized by selection of a covariate that split the sample into the most homogenous subsets of males and females. Therefore, the dependent variable in this analysis was a binary variable of males *versus* females. Gini coefficients provide a measure of impurity and indicate to what extent an independent variable splits the sample into homogenous sub-samples according to the dependent variable. CART is used in a wide range of applications, including public health, to identify interactive relationships and is often preferred over other recursive partitioning techniques because of its ease of interpretation [28].

**Figure 1:**
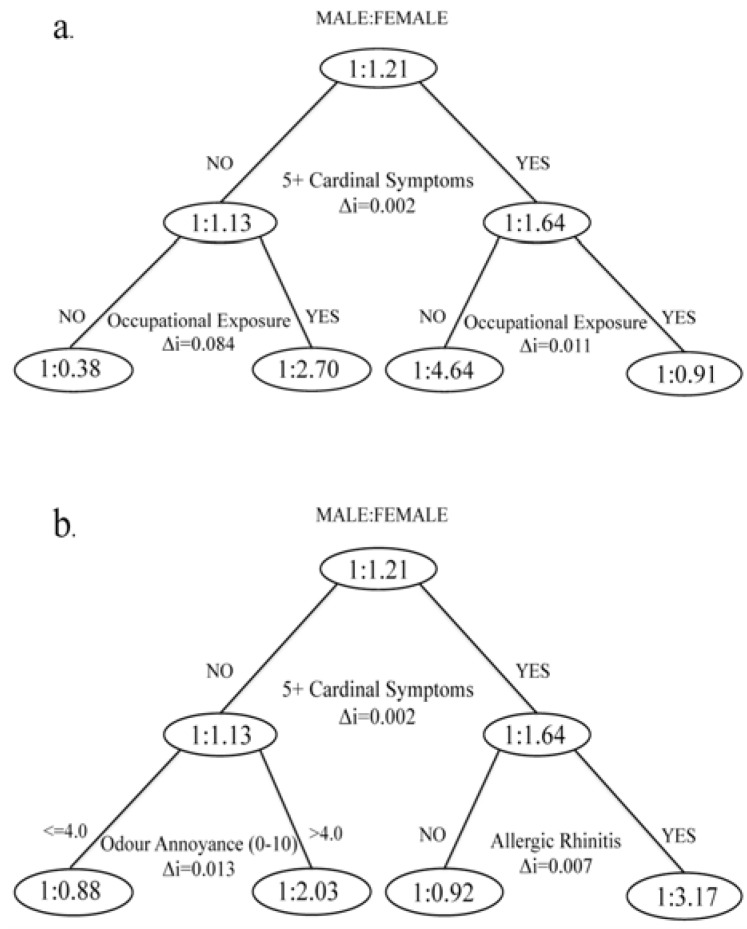
Classification and Regression Tree analysis for sex as dependent variable with 5+ cardinal symptoms forced as the first splitting variable. (**a**) Correctly classified = 71.3%; misclassification risk = 0.287 (SE = 0.015). (**b**) Occupational exposure removed from independent variables; correctly classified = 59.3%; misclassification risk = 0.407 (SE = 0.017).

Our covariates included 36 measures of co-exposure, health and lifestyle, socioeconomic characteristics, occupation, living conditions, health care use and access, social capital, family characteristics, community perceptions and health related behaviour. The maximum tree depth was set to 2 branches and the variable indicating 0–4 *vs*. 5+ CS was forced into the CART to provide the first split. Figure 1(a) shows that splitting the sub-samples that reported 0–4 or 5+ CS by exposure to gases, fumes or chemicals at work produced the most homogenous groups of males and females. Occupational exposure was consequently removed as a covariate and we repeated the analysis (Figure 1(b)), which revealed that spitting the 5+ CS sub-sample by respondents reporting hay fever or other allergies (except skin) produced the most homogenous groups of males and females.

### 2.4. Analysis

We built a series of logistic regression models that progressively included predictors of cardinal symptoms commonly reported in the literature. The models were fitted on the entire sample (n = 804) and sub-samples stratified on occupational exposure and allergic rhinitis. For the sample and sub-samples Model 1 is a bivariate model testing the influence of air pollution on 5+ CS. Model 2 is a multivariate model that test for the relative contribution of air pollution when controlling for the presence of indoor irritants (pets, carpets/rugs, and fireplace). Model 3 tests the effect of sex as a biological construct on cardinal symptoms, and Model 4 additionally controls for age. Model 5 includes a measure of income categorized as below or above the Statistics Canada Low Income Cut-Off [29] at $22,139 before tax (2005 LICO) for the median two household members in our sample and population size of Sarnia. Model 6 controls for chronic disease, including asthma, cancer, kidney disease, skin conditions, hypertension and heart disease.

Cases were weighted by direct age standardization to Sarnia proportions reported in the 2006 census to control for the confounding effects of age on cardinal symptoms, as the age group 65+ was overrepresented in our sample [30]. We report odds ratios (OR) generated by logistic regression to represent the relationships between the predictors and cardinal symptoms. We additionally report McFadden’s pseudo-*R^2^* values, the Hosmer & Lemeshow chi-square goodness of fit measure and the percentage correctly classified. All analyses were conducted with SPSS 18 and ArcGIS 9.3.

## 3. Results

### Sample Characteristics

After age standardization our sample (n = 804) represented a population with a median age of 49 and females were slightly over-represented at 54.3% compared to 52.3% in Sarnia [29]. However, females represented 54.7% of the sample before standardization. Bivariate tests of association showed that among the individual cardinal symptoms only nausea was reported significantly more often by females, but overall females reported 5+ CS more often before and after age standardization (Table 1). Women reported significantly higher rates of several chronic diseases, including skin conditions and allergies, while a higher proportion of males had heart disease. There were no differences in estimated air pollution exposure between the sexes (Table 1). Bivariate analyses also demonstrated a significant relationship between air pollution the dependent variable and several of the independent variables, specifically indicating that higher levels of exposure to a combination of NO_2_, SO_2_, and BTEX were associated with reporting 5+ CS, asthma and low income.

## 4. Cardinal Symptoms of Exposure

### 4.1. Complete Sample

Higher pollution rankings significantly increased the odds of reporting 5+ CS in Model 1 through 6 by 25%–35% (Table 2). The crude OR (Model 1) for the pollution factor was 1.28 and it increased as indoor exposure and sex were included, but decreased after controlling for age, low income and chronic diseases. The age groups 45–64 and 65+ were significantly less likely to report 5+ CS throughout the hierarchical models. This was due to the spatial distribution of age in our sample and Sarnia at large, as the highly polluted areas are populated with younger residents. In fact, there was a significant negative correlation between age and air pollution levels (Pearson’s *r* = −0.102, *p* < 0.05)*.* As expected, the likelihood of reporting 5+ CS was higher among asthmatics (OR: 3.99) and the hypertensive (OR: 1.94). Respondents who reported incomes below the LICO were 80% more likely to report 5+ CS when controlling for exposure, sex, age and chronic diseases. Females were approximately 50% more likely to report 5+ CS after controlling for exposure, age, low income and chronic disease.

### 4.2. Occupational Exposure Stratification

This series of models was fitted to sub-samples stratified by respondents who either were or were not, at the time or previously, exposed to gases, fumes or chemicals at work (Table 3). There were 432 respondents who reported no occupational exposure, of which 73.4% were female. As such, this sample was suited for inference regarding the influence of sex as a biological construct because controlling for occupational exposure also controlled for a substantial source of gendered co-exposure. The effect sizes for females were elevated in both sub-samples of occupational exposure, suggesting that gendered exposure patterns to airborne pollutants did not solely account for higher levels of 5+ CS reported among females in the complete sample.

**Table 2:** Odds ratios for 5+ cardinal symptoms—complete sample (n = 804).

	Model 1	Model 2	Model 3	Model 4	Model 5	Model 6
Pollution Factor	1.282 **	1.343 *	1.351 *	1.321 **	1.286 *	1.254 *
Indoor Exposure		1.181	1.187	1.192	1.225	1.286 *
Sex (54.3% female)			1.479 *	1.520 *	1.512 *	1.523 *
Age Group (Reference 18–24)				**	**	***
25–44				0.947	1.021	0.705
45–64				0.559 *	0.594	0.302 ***
65+				0.381 **	0.398 **	0.106 ***
Low Income					1.820 *	1.794 *
Asthma						5.048 ***
Cancer						1.765
Kidney Disease						2.973 **
Skin Condition						2.602 ***
Hypertension						2.426 ***
Heart Disease						1.724
Hosmer & Lemeshow **χ^2^ (**df), significance	16.71(8), 0.03	15.97(8), 0.43	1.09(8), 0.99	13.04(8), 0.11	6.17(8), 0.63	8.54(8), 0.38
Nagelkerke *R^2^*	0.014	0.019	0.028	0.058	0.067	0.207
% Correctly Classified	80.7	80.7	80.7	80.7	80.7	81.1

* *p* < 0.05; ** *p* < 0.01; *** *p* < 0.001.

Without the confounding effect of occupational exposure and chronic disease in Model 6, the pollution factor had a stronger effect on 5+ CS reporting compared to the complete sample (Table 3). Chi-square tests showed a significant relationship between 5+ CS reporting and asthma, skin conditions, hypertension, kidney disease and allergies, but marked differences in effects of the chronic diseases (excluding asthma) were observed within these sub-samples. Specifically, hypertension contributed to 5+ CS (OR: 2.9) reporting for respondents with occupational exposure, while kidney diseases and skin conditions contributed for respondents with no occupational exposure. The no occupational exposure sub-sample was characterized by 73.4% females and they reported higher levels of skin conditions, which may explain this finding, but the difference in effects of kidney disease among the sub-samples ins unclear.

Among respondents with no occupational exposure, indoor residential irritants significantly increased the likelihood of 5+ CS. The pollution factor was not a significant predictor among respondents with previous occupational exposure to dust, fumes and chemicals (Table 4). These findings corroborate that a combination of occupational, ambient residential, and indoor irritant exposure along with chronic diseases increased 5+ CS reporting in the complete sample. Interestingly, the effect of low income and age varied notably between the sub-samples. Older age groups with occupational exposure were significantly less likely to report 5+ CS, or in other words, younger people were more likely to report 5+ CS in this sub-sample. The regression diagnostics indicated that stratification by occupational exposure strengthened the models. The Nagelkerke *R^2^* value was higher in both sub-samples compared to the complete sample, and the percentage correctly classified was higher in the no occupation exposure sub-sample.

**Table 3:** Odds ratios for 5+ cardinal symptoms—no occupational exposure (n = 432).

	Model 1	Model 2	Model 3	Model 4	Model 5	Model 6
Pollution Factor	1.347 *	1.525 **	1.570 **	1.560 **	1.488 *	1.384
Indoor Exposure		1.535 **	1.570 **	1.577 *	1.679 **	1.773 **
Sex (73.4% female)			2.268 *	2.388 *	2.347 *	2.389 *
Age Group (Reference 18–24)						*
25–44				1.057	1.311	0.953
45–64				0.827	1.009	0.576
65+				0.535	0.639	0.178 **
Low income					2.675 *	2.617 *
Asthma						5.173 ***
Cancer						1.883
Kidney Disease						3.348 *
Skin Condition						3.818 ***
Hypertension						1.964
Heart Disease						2.924
Hosmer & Lemeshow **χ^2^ (**df), significance	8.98(8), 0.34	5.29(8), 0.73	5.37(8), 0.72	12.41(8), 0.13	5.63(8), 0.69	11.30(8), 0.19
Nagelkerke *R^2^*	0.018	0.047	0.07	0.082	0.106	0.267
% Correctly Classified	85.3	85.3	85.3	85.3	85.3	85.9

* *p* < 0.05; ** *p* < 0.01; *** *p* < 0.001.

**Table 4:** Odds ratios for 5+ cardinal symptoms—occupational exposure (n = 372).

	Model 1	Model 2	Model 3	Model 4	Model 5	Model 6
Pollution Factor	1.280	1.257	1.260	1.191	1.162	1.194
Indoor Exposure		0.931	0.887	0.879	0.891	0.948
Sex (26.6% female)			2.369 ***	2.316 ***	2.381 ***	2.327 **
Age Group (Reference 18–24)				**	**	***
25–44				0.576	0.583	0.393 *
45–64				0.288 **	0.285 **	0.132 ***
65+				0.242 **	0.232 **	0.051 ***
Low Income					1.802	2.085
Asthma						5.622 ***
Cancer						1.898
Kidney Disease						2.500
Skin Condition						1.916
Hypertension						2.924 **
Heart Disease						1.129
Hosmer & Lemeshow **χ^2^ (**df), significance	16.79(8), 0.03	11.20(8), 0.19	8.84(8), 0.36	12.41(8), 0.13	11.27(8), 0.18	9.18(8), 0.33
Nagelkerke *R^2^*	0.015	0.016	0.063	0.115	0.122	0.248
% Correctly Classified	75.3	75.3	75.3	75.3	75.3	75.9

* *p* < 0.05; ** *p* < 0.01; *** *p* < 0.001.

### 4.3. Allergic Disease Stratification

These regression models were fitted on sub-samples stratified by respondents who reported having allergic diseases except for skin. The sub-sample that reported no allergies represented 572 respondents, of which 49.6% were female. The results of this analysis as presented in Table 4 shows notable differences from the complete sample with respect to effects of sex, age and indoor irritants. We observed a higher likelihood of reporting 5+ CS by respondents below the LICO reporting, but only within the sub-sample with no allergies or hay fever. Most notable is the change in effect of the pollution factor, which disappears altogether among respondents with allergies and only appears as a significant covariate of indoor exposure and sex among respondents with no allergies (Table 5). Removing respondents with allergies from the analysis also reduces the effect of air pollution when controlling for age.

**Table 5:** Odds ratios for 5+ cardinal symptoms—no allergies or hay fever (n = 572).

	Model 1	Model 2	Model 3	Model 4	Model 5	Model 6
Pollution Factor	1.241	1.356 *	1.356 *	1.284	1.237	1.211
Indoor Exposure		1.390 *	1.388 *	1.421 *	1.500 **	1.577 **
Sex (49.6% female)			0.938	0.964	0.938	0.910
Age Group (Reference 18x2013;24)				***	***	***
25x2013;44				0.506 *	0.576	0.442 *
45x2013;64				0.215 ***	0.238 ***	0.135 ***
65+				0.288 **	0.314 **	0.102 ***
Low Income					2.386 **	2.764 **
Asthma						3.422 **
Cancer						1.607
Kidney Disease						1.152
Skin Condition						3.895 ***
Hypertension						2.453 **
Heart Disease						2.072
Hosmer & Lemeshow **χ^2^ (**df), significance	8.07(8), 0.43	5.76(8), 0.76	9.56(8), 0.30	3.41(8), 0.91	9.99(8), 0.27	9.88(8), 0.27
Nagelkerke *R^2^*	0.01	0.028	0.028	0.09	0.108	0.215
% Correctly Classified	85.9	85.9	85.9	85.9	85.9	85.9

* *p* < 0.05; ** *p* < 0.01; *** *p* < 0.001.

Model 6 in Table 6 explains more variation in 5+ CS reporting than any other model (*R^2^* = 0.32). Within this sub-sample that reported having allergies, odds ratios for reporting 5+ CS were 2.7 for females, 6.3 for asthmatics, and 11.5 for respondents with kidney disease. The effect size of asthma was notably reduced when we excluded respondents who reported hay fever and allergies from our sample. This was expected because allergic reactions commonly include the lower respiratory tract. Conversely, asthmatics and respondents with kidney disease were more likely to report 5+ CS in the allergy sub-sample. We observed no sex differences in reporting asthma or kidney disease, and females in the no allergies sub-sample did not have an increased likelihood of reporting 5+ CS in Model 1 through 6. Given the strong correlation between our dependent variable and the pollution factor, this result suggests that higher rates of allergic diseases among women may have increased the susceptibility to short-term effects of air pollution observed in the complete sample.

**Table 6:** Odds ratios for 5+ cardinal symptoms—allergies or hay fever (n = 232).

	Model 1	Model 2	Model 3	Model 4	Model 5	Model 6
Pollution Factor	1.354	1.310	1.355	1.287	1.273	1.279
Indoor Exposure		0.899	0.904	0.845	0.850	0.891
Sex (50.4% female)			2.212 *	2.272 *	2.266 *	2.754 **
Age Group (Reference 18–24)				**	**	**
25–44				3.667 *	3.724 *	2.310
45–64				3.250	3.258	1.473
65+				0.915	0.909	0.179 *
Low Income					1.382	1.587
Asthma						6.305 ***
Cancer						2.728
Kidney Disease						11.493 **
Skin Condition						1.383
Hypertension						2.019
Heart Disease						0.895
Hosmer & Lemeshow **χ^2^ (**df), significance	8.88 (8), 0.35	6.85 (8), 0.55	12.89 (8), 0.12	3.20 (8), 0.92	8.22 (8), 0.41	5.27 (8), 0.73
Nagelkerke *R^2^*	0.023	0.025	0.063	0.138	0.140	0.322
% Correctly Classified	67.8	67.8	67.8	67.8	67.8	73.8

* *p* < 0.05; ** *p* < 0.01; *** *p* < 0.001.

## 5. Discussion

Overall, the study confirmed previous findings that demonstrated the effects of demographics, SES and health covariates on short-term effects of air pollution. We observed that women were approximately 50% more likely than men to report five or more cardinal symptoms of exposure to a combination of NO_2_, SO_2_, and BTEX. There is, however, an important distinction between this study and other work on short-term pollution effects. Our outcome measurement provided a much broader indication of potential pollution health effects than studies looking at mortality or hospitalization, although we also observed elevated probabilities of reporting cardinal symptoms by those traditionally thought to be at higher risk. The analysis showed that both biological and cultural differences between men and women were influential in predicting symptoms of pollution exposure.

We found that females had an increased susceptibility to 5+ CS reporting when they were also dealing with allergic diseases. Females had higher rates of allergic disease in our sample. Although working with female adolescents, Fagan and others also reported higher rates of allergies among females [31]. Previous findings s on the susceptibility to air pollution among allergy sufferers remain equivocal [10,32], though children with allergies can have more severe asthma due to air pollution [33]. There are genetic differences between sexes that influence inflammatory responses to allergens [34], while sex hormones can affect the immune system and cause chemical hypersensitivity [35]. These findings provide biological plausibility for the potentiation of air pollution effects in females being due to allergic disease.

Women in our study also had a significantly higher rate of skin conditions, and the prevalence of eczema is associated with air pollution even in areas with relatively low concentrations [36]. Previous research found that females report skin conditions such as hand eczema more often, and most female-dominated occupations require wet work and are more likely to cause irritant contact dermatitis [11]. Kreutzer *et al*. [37] found that females reported chemical sensitivity more often, but also that both males and females believed they were made sick by common chemical exposures. The possibility for psychosomatic effects therefore exists, but more importantly our findings support previous research that found women to (correctly) perceive air pollution as a higher risk to their health than men [38]. Taken together these findings suggest that complex interactions between gendered co-exposures at work, sex-related determinants of vulnerability and ambient pollution exposure profiles can confound health effects of air pollution.

We found that occupational exposure modified the associations between chronic diseases and cardinal symptom reporting. Specifically, skin conditions and kidney diseases were a strong predictor of 5+ CS in the sub-sample with no occupational exposure *versus* hypertension having a strong effect among occupationally exposed. Interestingly, certain kidney diseases and atopic disorders are associated [39]. The difficulty of determining the moderating effects of occupational co-exposure was illustrated by a re-analysis of data for The Six Cities Study of mortality associated with PM_2.5_, which found that the effect of air pollution was not consistently different in individuals with “dirty jobs” and exposure to lung carcinogens at work [40]. We found that the effects of air pollution were non-significant among people who reported occupational exposure and highly significant among respondents with no occupational exposure. This does not, however, suggest that people with occupational exposure are less susceptible to effects of air pollution, but rather highlights the potentially confounding effects of aggregate exposure assessment and the importance of controlling for co-exposures in industrial cities like Sarnia [16].

The relationship between hypertension and cardinal symptom reporting as shown in our analysis is complicated because air pollution can cause both hypertension [41] and cardinal symptoms independently; several of our outcome indicators, including nausea and trouble breathing, are also side effects of antihypertensive drugs; and chronic disease that should not be associated with air pollution may cause people to report excessive air pollution effects despite no differences in exposure compared to healthy people [42]. Furthermore, Clougherty and colleagues [43] reported higher rates of hypertension among women working in manufacturing jobs, and we found that hypertension was a strong predictor of cardinal symptom reporting among those occupationally exposed. Sex-related responses to workplace hazards and gender-related differences in work status are important considerations in this context. Therefore, and similar to the current study, Clougherty *et al*. [43] recognized the need to separate sex and gender effects on health when investigating the elevated health risks among women in manufacturing jobs as measured by onset of hypertension. They stratified the male and female sub-samples by propensity scores calculated from *a priori* effects of gender on job status (hourly *versus* salaried). The propensity scores were utilized to control for pre-hire gendered effects on job status, such as socioeconomic factors. They observed higher rates of hypertension only among women predicted to be hourly workers, which suggests that vulnerabilities are gendered in addition to potentially being sex-related. Unlike the current study they lacked information on chemical exposures, so it was not known whether sex-related responses to workplace hazards were due to differing exposures between men and women (which we try to show) or, for example, anatomical differences affecting safety equipment effectiveness.

The analytical approach utilized by Clougherty *et al*. [43] is a confirmatory, hypothesis-testing alternative to the technique utilized in the current study, which was to use CART to identify covariates that interacted with the binary construct male/female, and furthermore control for these covariates by stratification. To our knowledge no other study has utilized CART to investigate sex and gender effects on health outcomes related to air pollution specifically, but recursive partitioning methods have been used in other environmental health studies. For example, this methodology was used to identity subgroups with similar time-activity relationships to Volatile Organic Compound (VOC) exposure in the Exopolis study [44]. Keegan *et al*. [45] used recursive partitioning to identify associations between the built environment and physical health, and Hu *et al*. [46] investigated the interactive effects of ambient temperature and sulphur dioxide on total mortality in Sydney, Australia by developing a time-series CART model. These studies demonstrate the utility of CART and similar techniques as an exploratory tool that can be used to identify variables of interest or interactions between specific predictor variables. The use of this technique is more widespread in clinical and diagnostic studies, but our application here suggests there may be opportunities for advancing its use in environmental health research. As applied to sex, gender and health research, recursive partitioning models offer a viable alternative to previously proposed analytical approaches, such as propensity analyses and multi-level modeling [1], when lack of longitudinal data or study design does not permit using these techniques. Furthermore, CART can assist researchers to disentangle the effects of biology and culture in community-based studies where contextual factors may be influential.

Forastiere *et al*. [47] argues that short-term effects of air pollution on mortality are mainly due to pre-existing health conditions that increase susceptibility. The cross-sectional study design meant we were not able to investigate the etiology of chronic disease in our respondents, but other research has shown that prolonged exposure to air pollution can lead to asthma [48]. One of the cardinal symptoms was wheezing or trouble breathing, so not surprisingly asthmatics were more likely to report 5+ CS.

Our results relating to different age groups and the relationship between sex, cardinal symptoms and air pollution in Sarnia should be interpreted with caution because of an inverse correlation between age and residential exposure, but a study conducted in Sweden found that the health-related quality of life was reduced by rhinitis and asthma more so in women than men aged 50–64 [49]. Varying prevalence of pre-existing health conditions that increase susceptibility introduces another level of uncertainty to the modifying effects of occupational co-exposure on air pollution health outcomes. It is possible that socioeconomic or physiological determinants of chronic disease also influence selection of types of work that involve exposure to chemicals. In this study it was not possible to determine whether differences in susceptibility among occupationally exposed were due to sex, gender, or both, due to the cross-sectional study design. Nevertheless, the overall results support calls [1,50] for sex, gender and health considerations in environmental exposure research.

## 6. Conclusions

Butter suggests that environmental health studies pay particular attention to risk disparities among males and females because some autoimmune disorders show strong sex differences, and furthermore environmental exposures are known risk factors for some of these disorders [50]. This study found that measuring cardinal symptoms in potentially exposed populations may provide a tool to identify those at higher risk from air pollution due to autoimmune disorders, and in our sample this population was predominantly female. Furthermore, we show that identifying and controlling for influential covariate interactions by stratification in air pollution studies can be helpful in teasing out gender and sex effects where exposure assessment is limited to residential location. Simply controlling for occupational exposure indicated that in addition to preexisting conditions, sex differences in these conditions and gendered co-exposure are important determinants of susceptibility to air pollution. The study was strengthened by a representative sample from a community with significant differences in exposure among respondents. However, the influence of occupational exposure on our models of air pollution health effects suggests that more information about daily movement within the city and consequent variability in exposure among individuals or groups would have been beneficial. Future studies on sex and gender effects on environmental health outcomes should consider using an exposure measure that reflects differences in occupational and leisure activities along with residential estimates.
